# Benefits of Probiotic Pretreatment on the Gut Microbiota and Minor Complications after Bowel Preparation for Colonoscopy: A Randomized Double-Blind, Placebo-Controlled Pilot Trial

**DOI:** 10.3390/nu15051141

**Published:** 2023-02-24

**Authors:** Dooheon Son, Youn Jung Choi, Min Young Son, Won Moon, Seun Ja Park, Sanghyun Lim, Jae Hyun Kim

**Affiliations:** 1 Cell Biotech, Co., Ltd., Gimpo-si 10003, Republic of Korea; 2 Department of Internal Medicine, Kosin University College of Medicine, Busan 49267, Republic of Korea

**Keywords:** probiotics, gut microbiota, bowel preparation, colonoscopy, complication

## Abstract

The aim of this study was to evaluate the effects of probiotic pretreatment on the alteration and recovery of gut microbiota after bowel preparation and its correlation with minor complications. This was a randomized, double-blind, placebo-controlled pilot trial that included participants 40–65 years of age. Participants were randomly provided probiotics (active group) or placebo (placebo group) for 1 month before the colonoscopy and their feces collected. A total of 51 participants were included in the present study (26 in the active group and 25 in the placebo group). In the active group, the microbial diversity, evenness, and distribution were not significantly changed between before and after bowel preparation, but did change in the placebo group. The number of gut microbiota that decreased after bowel preparation in the active group was lower than in the placebo group. On the seventh day after colonoscopy, the gut microbiota in the active group was restored to almost the same level as before bowel preparation. In addition, we identified that several strains were assumed as key microbiota in early colonization and some taxa were increased only in the active group after bowel preparation. In multivariate analysis, taking probiotics before bowel preparation was identified as a significant factor for decreasing the duration of minor complications (odds ratio 0.13, 95% confidence interval 0.02–0.60, *p* = 0.027). Probiotic pretreatment had benefits on the alteration and recovery of gut microbiota and possible complications after bowel preparation. Probiotics may also aid in the early colonization of key microbiota.

## 1. Introduction

Colonoscopy is an effective screening tool to reduce the incidence of colorectal cancer by detecting and removing colorectal polyps. However, the adherence rate to screening colonoscopy remains low; the overall participation rate for screening colonoscopy was only 38% compared with 67% for a fecal occult blood test [1,2]. Patient-reported barriers to screening colonoscopy included concerns regarding bowel preparation and fear of pain [3]. Colonoscopy can be associated with serious complications of perforation and hemorrhage as well as minor complications including abdominal discomfort, bloating, nausea/vomiting, diarrhea, and constipation [4,5]. The occurrence of these complications can affect the patient’s satisfaction and result in their refusal of future colonoscopies. The incidence of minor complications after colonoscopy is reportedly between 24% and 32%, occurring within 7 days after colonoscopy [5,6]. In a recent multicenter study of 793 subjects, 361 (45.5%) complained of newly developed symptoms including epigastric/abdominal bloating, pain, and dyspepsia after colonoscopy. Female sex and history of inflammatory bowel disease were significantly associated with development of these symptoms [7].

Emerging evidence showed that dysbiosis of the gut microbiome was found in various conditions, including metabolic diseases, inflammatory diseases, and cancers. In several studies, bowel preparation for colonoscopy was shown to change the gut microbial diversity, composition, and metabolome, and these effects could last up to 1 month [8,9,10,11]. To minimize these effects, bowel preparation using two separate dosages has been proposed, which causes fewer alterations to the intestinal microbiota than the single dose [12]. In our previous study, five of 24 healthy subjects experienced minor complications after colonoscopy and most were mild and self-limited. In addition, we showed that patients with minor complications after bowel preparation have a higher *Firmicutes*/*Bacteroidetes* ratio in the initial stool samples collected before bowel preparation, and the ratio decreased after bowel preparation [13]. Furthermore, we performed a preliminary study with 100 volunteers without age restriction to evaluate the efficacy of 1-month probiotics after bowel preparation (open-label trial; 50 people received probiotics and 50 people did not). Results showed that the gut microbial diversity sharply decreased after bowel preparation and the changed microbiota was almost restored on the 21st day after bowel preparation regardless of probiotics [14].

Based on these results, we hypothesized that the minor complications after colonoscopy were associated with an initial gut microbial status of the subject, and that the modification of this microbial status before bowel preparation can help reduce the occurrence of complications after colonoscopy. The aim of this study was to investigate the beneficial effects of probiotic pretreatment on the alteration and recovery of gut microbiota after bowel preparation for colonoscopy and its association with minor complications.

## 2. Methods

### 2.1. Study Design and Participants

**Figure 1:**
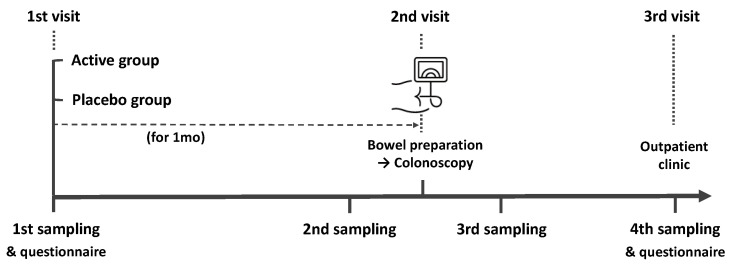
Schematic drawing of the time flow used in this study.

The present study was a randomized, double-blind, placebo-controlled pilot trial. We recruited healthy participants aged 40–65 years undergoing colonoscopy for screening or post-polypectomy surveillance. The exclusion criteria in our study were as follows: history of cancer, inflammatory bowel disease, lactose intolerance, or obesity (body mass index (BMI) > 30), and use of antibiotics, laxatives, metoclopramide, tegaserod, erythromycin, or proton-pump inhibitors within 1 month before colonoscopy. A study investigator explained in detail the aim and contents of the study to the participants and all subjects provided written informed consent. Information including age, sex, height, body weight, BMI, smoking and alcohol history, co-morbidities, the reason for a colonoscopy, previous colonoscopy experience, symptoms before colonoscopy, dietary habits, the number of defecations per day or week, and Bristol Stool Scale (BSS) were collected using a questionnaire. The study outline is summarized in Figure 1. All participants collected their feces 4 times (1 month before, 2 to 3 days before, 1 to 2 days after, and 7 days after colonoscopy) using a Fecal Swab kit (Noble Biosciences, Gyeonggi-do, Korea) that could be stored at room temperature. Participants were randomly provided probiotics or placebo 1 month before the colonoscopy. All patients were provided an educational brochure on bowel preparation and colonoscopy, including a diet schedule (low-fiber diet for 3 days and soft diet for dinner the day before the colonoscopy). They ingested 1 L polyethylene glycol solution with ascorbic acid between 7 and 9 PM the day before the colonoscopy and the remaining 1 L the next morning at least 2 h before the colonoscopy. During the colonoscopy, bowel preparation scale was assessed and polyps < 1 cm were removed. On the 7th day after colonoscopy, a questionnaire was administered to participants regarding any complications. The questionnaire included the type, timing, frequency, and duration of complications and was used to evaluate the occurrence of minor or severe complications after colonoscopy. The local institutional review board evaluated and approved the present study protocol (KUGH 2021-01-025) and this trial was registered with the International Clinical Trials Registry Platform (no. KCT0006321).

### 2.2. Randomization

Participants who met the inclusion criteria were randomly assigned to either the active or placebo group. Randomization was performed using a computer-generated randomization list by a study investigator and the list was placed into sealed opaque envelopes that were not opened until the end of the study. The active group was given the package of probiotics (1.0 × 10^10^ colony-forming units/5 g per day) consisting of *Lactobacillus. acidophilus CBT LA1*, *Lactobacillus rhamnosus CBT LR5*, *Bifidobacterium lactis CBT BL3*, *Bifidobacterium longum CBT BG7*, *Bifidobacterium bifidum CBT BFs*, and *Streptococcus thermophilus CBT ST3* for 1 month, and the placebo group was given a package of maltodextrin (same properties, formulation, weight, and volume as the probiotics) for 1 month. For blind control, these products were packaged with unprinted paper; therefore, participants did not know which group they belonged to until the end of the study.

### 2.3. Endpoints

The primary outcome was to evaluate the effect of probiotic pretreatment on the alteration and recovery of gut microbiota after bowel preparation for colonoscopy. In addition, the secondary outcomes were to assess the effect of probiotic pretreatment on the occurrence of minor complications after colonoscopy and to determine which microbiome plays an important role in the colonization at the early stage after bowel preparation.

### 2.4. Sample Preparation and Data Analysis

The collected samples were transported to Cell Biotech, Co., Ltd. (Gyeonggi-do, Korea) and immediately frozen at −80 °C. A total of 204 samples were used for microbiome analysis. Microbial DNA was extracted using the FastDNA SPIN Kit for Soil (MP Biochemicals, Santa Ana, CA, USA) according to the manufacturer’s instructions. The extracted microbial DNA was purified using DNeasy PowerClean Cleanup Kit (Qiagen, Hilden, Germany) and then DNA quality was measured using NanoDrop (Thermo Fisher Scientific, Carlsbad, CA, USA). The purified DNA was measured for DNA concentration using the Qubit™ dsDNA BR Assay kit (Thermo Fisher Scientific). A sequencing library was prepared according to the Illumina 16S Metagenomic Sequencing Library Preparation Guide (Illumina, San Diego, CA, USA). The V4-V5 region of the bacterial 16S rRNA gene was amplified for 16S rRNA gene sequencing. The forward primer in the v4 region (CCA GCM GCC GCG GTA ATW C) and the reverse primer in the v5 region (CC GTC AAT TYY TTT RAG TTT) were used for PCR amplification in this study. The amplified sequencing library was purified with Agencourt^®^ AMPure XP beads (Beckman Coulter, Brea, CA, USA) and the quality of the library was confirmed using a 2100 Bio-analyzer (Agilent, Santa Clara, CA, USA). The library pool was sequenced with 250 bp paired-end reads on the MiSeq platform using the MiSeq reagent kit V2 (Illumina).

### 2.5. Statistical Analysis

Raw sequencing data were processed using Quantitative Insight into Microbial Ecology software package 2 (QIIME 2, v 2021.11, http://qiime2.org (accessed on 6 April 2022)). Denoising was performed using DADA2 and a taxonomy table was created using SILVA database (v138). Normalization to depth of 30,000, the minimum depth of the sample, was used for alpha and beta diversity analyses. Data visualization was performed using the ggplot package of R (v4.1.3) and statistical analysis was performed with Wilcoxon signed rank test, Kruskal–Wallis test, and PERMANOVA using the vegan package. Linear discriminant analysis (LDA) effect size analysis was performed using Galaxy (https://huttenhower.sph.harvard.edu/galaxy).

Continuous data with normal distributions are expressed as mean ± standard deviation, and categorical data are presented as the number of subjects (%). Student’s *t*-test and the chi-square test were performed for continuous and categorical variables, as appropriate. To analyze the factors associated with complications after colonoscopy, univariate and multivariate logistic regression analyses were performed. *p* values of <0.05 were considered statistically significant. Statistical analyses were performed using R (v4.1.3).

## 3. Results

### 3.1. Baseline Characteristics and Colonoscopy Outcomes

Between May 2021 and February 2022, a total of 52 participants signed written informed consent forms and were randomized to either the active or placebo group. After randomization, one subject was excluded due to consent withdrawal. Finally, 51 participants were included in the analysis. The mean age of participants was 53.7 ± 1.1 years and 32 (62.7%) were female. In both groups, height, weight, BMI, alcohol and smoking history, co-morbidities, dietary habits, stool frequency, stool consistency, reason for colonoscopy, previous experience of colonoscopy and polypectomy, bowel preparation, findings of colonoscopy, detected colon polyp size, and resection method of colon polyp were not significantly different. The baseline characteristics and colonoscopy outcomes are summarized in Table 1.

### 3.2. Alteration and Restoration of Gut Microbiota after Bowel Preparation

**Figure 2:**
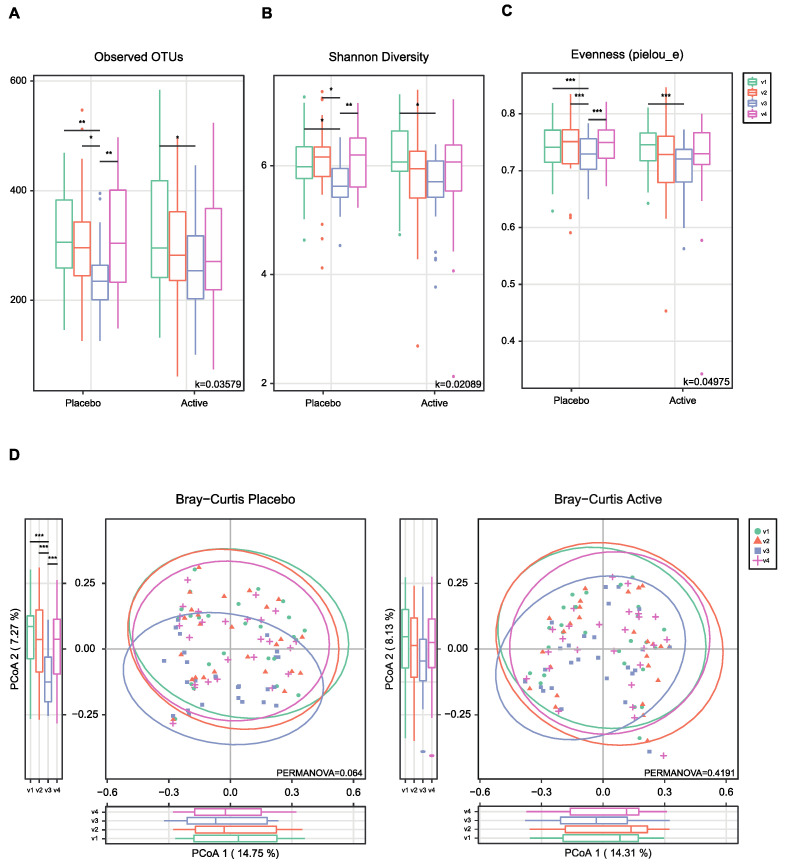
Alpha- and beta-diversity comparisons of the gut microbiomes of each group. (**A**) Boxplot of the Observed OTUs of each group. (**B**) Boxplot of the Shannon diversity of each group. (**C**) Boxplot of the Pielou’s evenness of each group. (**D**) Bray–Curtis distances within each group. The color of the boxplots and dots represents the different groups analyzed based on the legend (v1, visit1; v2, visit2; v3, visit3; v4, visit4). * *p* < 0.05, ** *p* < 0.01, *** *p* < 0.001 (Wilcoxon test). OTUs, operational taxonomic units.

**Figure 3:**
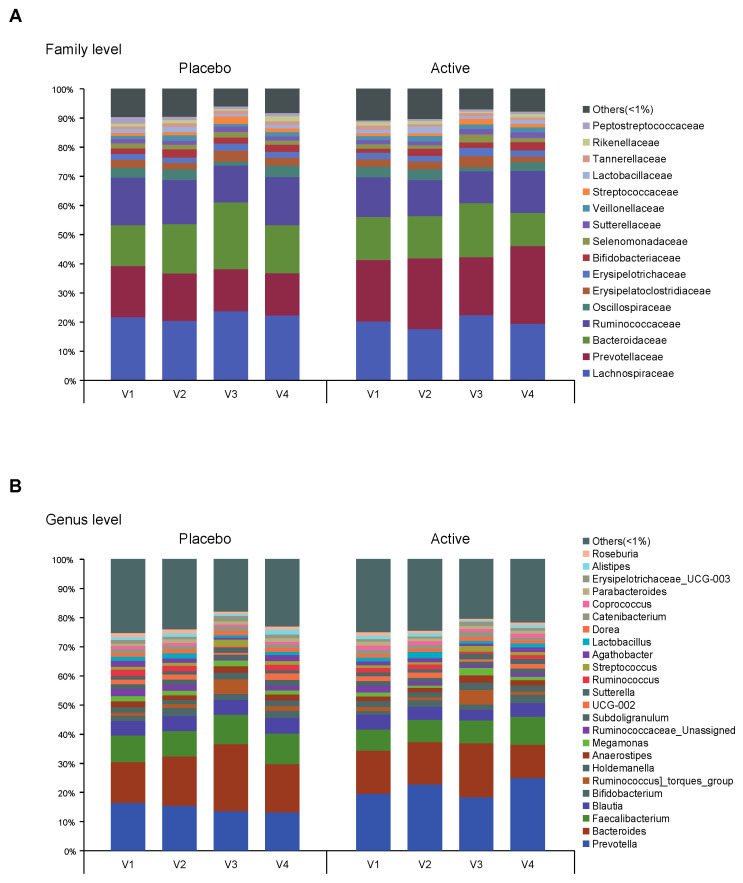
Taxonomy composition of each group. Bar plots on relative abundances of OTUs annotated at the (**A**) family and (**B**) genus level. An average <1% is labeled as others. Each bar represents the different group (v1, visit1; v2, visit2; v3, visit3; v4, visit4). OTUs, operational taxonomic units.

As shown in Figure 1, the feces were collected four times from participants and the microbial diversity was compared between the probiotics and placebo groups. At first, we evaluated the alpha and beta diversity at each time point (visit 1 (v1), 1 month before colonoscopy; v2, 2 to 3 days before; v3, 1 to 2 days after; v4, 7 days after) in both groups. The observed operational taxonomy units (OTUs) and Shannon diversity were significantly decreased after bowel preparation (v3) compared to the baseline (v1) in both groups. These measures significantly decreased after bowel preparation (v3) compared to 2 or 3 days before bowel preparation (v2) only in the placebo group (Figure 2A,B). Evenness also significantly decreased after bowel preparation (v2–v3) only in the placebo group (Figure 2C). In the Bray–Curtis analysis, the microbial distribution showed a different tendency before and after bowel preparation (v2–v3) in the placebo group (Figure 2D), although it was not statistically significant. In the assessment of taxonomy composition, an increase or decrease was observed in certain microbiota at the family and genus levels after bowel preparation in both groups (Figure 3A,B).

**Figure 4:**
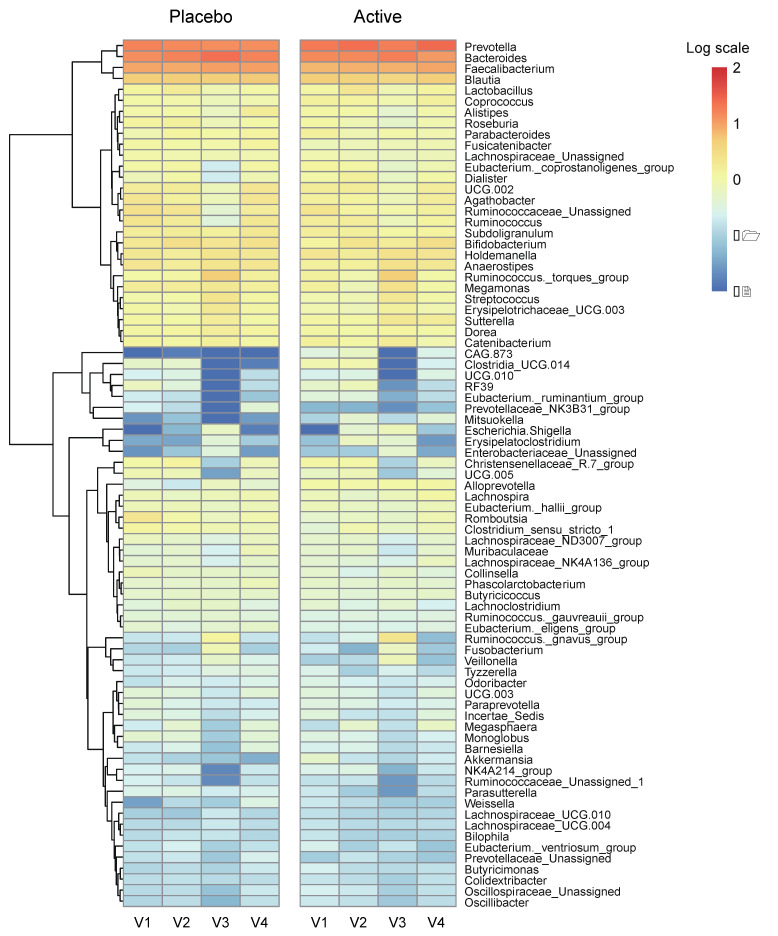
Heatmap analysis via relative abundance at the genus level. Heatmap on relative abundances of annotated OTUs at the genus level. Genus with an average < 0.01% were excluded. Ranges were applied to the analysis by logarithmic scale. OTUs, operational taxonomic units.

**Figure 5:**
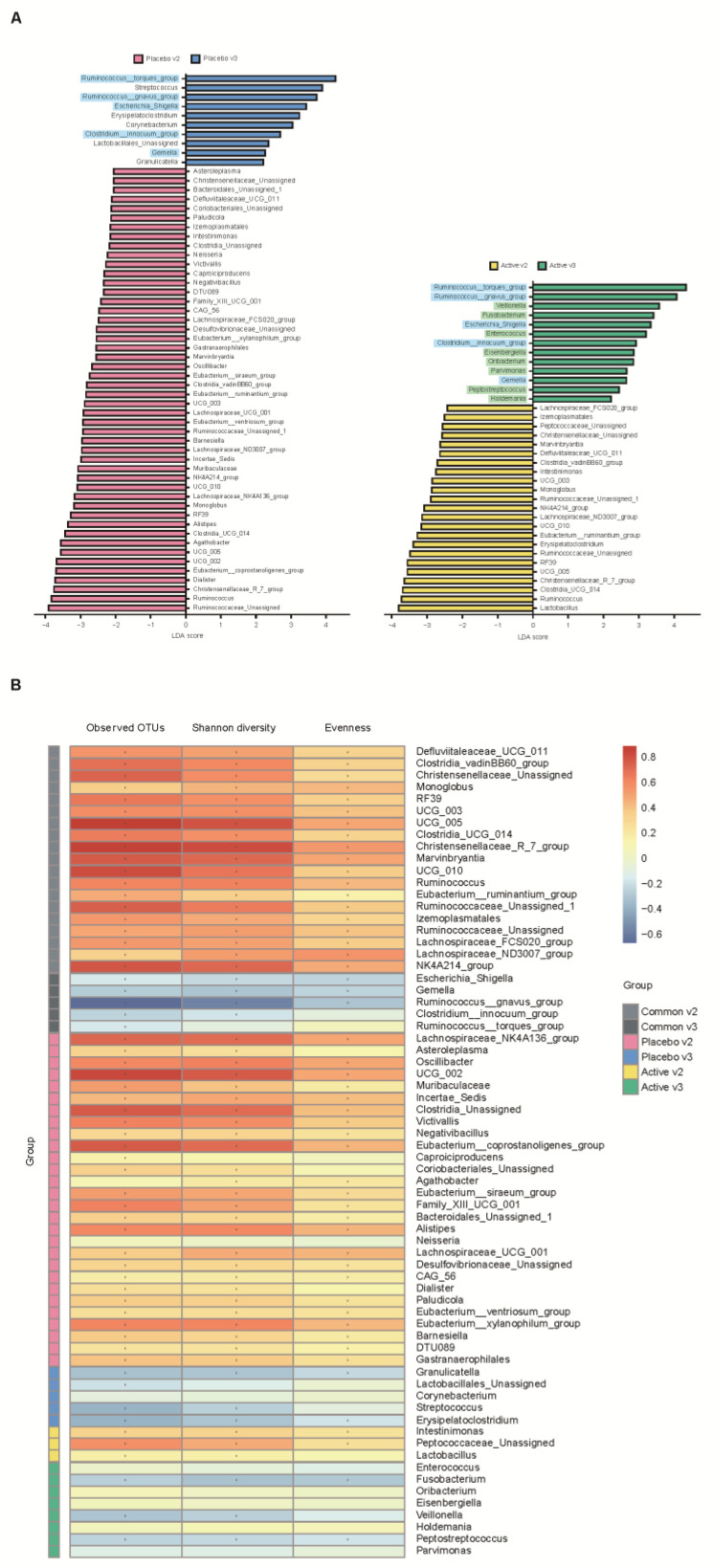
Comparative analysis of the 2nd and 3rd visit groups. (**A**) Microbiome analysis via linear discriminant analysis (LDA) effect size (LEfSe) at the genus level. The bar size represents the effect of specific taxa number in the particular group at the genus level. The alpha value for the factorial Kruskal–Wallis test was <0.05 and the threshold on the logarithmic LDA score for discriminative feature was >2.0. (**B**) Heatmaps indicate positive (red) and negative (blue) correlations between the alpha diversity and the taxa sorting in the results of LEfSe. The figure is drawn by sorting the taxa based on the result in (**A**). * *p* < 0.05 (Spearman’s correlation).

**Figure 6:**
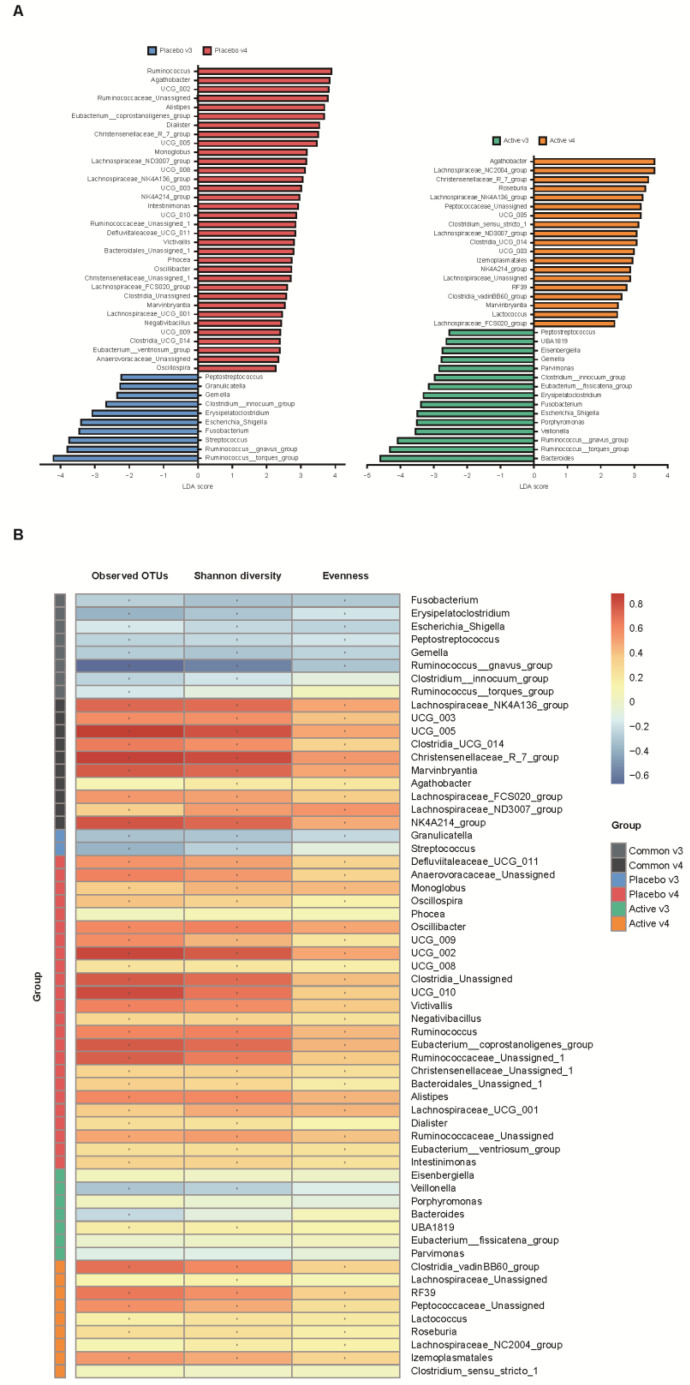
Comparative analysis of the 3rd and 4th visit groups. (**A**) Microbiome analysis via linear discriminant analysis (LDA) effect size (LefSe) at the genus level. The bar size represents the effect of specific taxa number in the particular group at the genus level. The alpha value for the factorial Kruskal–Wallis test was <0.05 and the threshold on the logarithmic LDA score for discriminative feature was >2.0. (**B**) Heatmaps indicate positive (red) and negative (blue) correlations between the alpha diversity and the taxa sorting in the results of LEfSe. The figure was drawn by sorting the taxa based on the result in (**A**). * *p* < 0.05 (Spearman’s correlation).

We conducted a heatmap analysis to identify the overall change of microbiota at each time point in both groups and identified that more taxa decreased after bowel preparation (placebo v3) and remained in decreased status on the 7th day after colonoscopy (placebo v4) in the placebo group compared to the active group (active v3 and v4) (Figure 4). Subsequently, the change in specific gut microbiota before and after bowel preparation was assessed using LDA effect size analysis. As shown in Figure 5A and Figure S1A, the number of increased gut microbiota after bowel preparation was similar in both groups (placebo v3 and active v3) at the genus and family levels. However, gut microbiota was abundant before bowel preparation (v2), and the decrease in microbiota after bowel preparation was larger in the placebo group than in the active group (placebo v2 and active v2). In addition, all decreases in microbiota in the active group (active v2) also were observed in the placebo group (placebo v2). On the 7th day after colonoscopy, the number of gut microbiota was higher in the placebo group (placebo v4) than in the active group (active v4) (Figure 6A and Figure S1B). In addition, the gut microbiota in the active group was restored to almost the same level as before bowel preparation. However, a few taxa including *Gastranaerophilales* and *Clostridia_UCG_014* remained decreased in the placebo group on the 7th day after colonoscopy (Figures S1C and S2).

### 3.3. Complications after Bowel Preparation and Factors Associated with Complications

On the 7th day after colonoscopy, a total of 17 participants answered that they had experienced non-serious complications. As shown in Table 2, the number of participants who had any complication after colonoscopy was similar in both groups; nine in the active group and eight in the placebo group. The type, onset timing, and frequency of complications were not different between the two groups. The duration of complications was typically ≤30 min in the active group; however, complications persisted for 1–2 h in three participants and >24 h in four participants in the placebo group (*p* = 0.025). The severity score of complications using the visual analogue scale in the placebo group was higher than in the active group but without statistical significance (4.1 ± 2.6 vs. 3.1 ± 1.6, *p* = 0.349).

### 3.4. Specific Gut Microbiota Observed in the Early Stage after Bowel Preparation and Correlation with Microbial Diversity

After bowel preparation (v3), several taxa including *Ruminococcus_torques_*group, *Ruminococcus_gnavus_*group, *Escherichia_Shigella*, *Clostridium_innocuum_*group, and *Gemella* were equally increased in both groups (blue highlight in Figure 5A) and then decreased on the 7th day after colonoscopy (v3 in Figure 6A). In addition, some taxa, including *Veillonella*, *Fusobacterium*, *Enterococcus*, *Eisenbergiella*, *Oribacterium*, *Parvimonas*, *Pepstreptococcus*, and *Holdemania*, were increased only in the active group after bowel preparation (green highlight in Figure 5A).

In addition, the relationship between alpha diversity/evenness and genus was evaluated. As shown in Figure 5B, the taxa positively correlated with alpha diversity and evenness decreased after bowel preparation (common v2–v3). Taxa positively correlated with greater decreased in alpha diversity in the placebo group (placebo v2–v3) compared to the active group (active v2–v3). On the 7th day after colonoscopy, the taxa positively correlated with alpha diversity increased in both groups (active v3–v4 and placebo v3–v4), and the number of increased taxa was higher in the placebo group than in the active group (Figure 6B).

## 4. Discussion

Despite the increasing evidence that gut microbiota are important in health and disease, the association between alteration of gut microbiota and bowel preparation for colonoscopy is underinvestigated. The results of the present study provide evidence for the beneficial effects of probiotic pretreatment on the alteration and recovery of the gut microbiome and possible complications after colonoscopy.

Probiotics are live microorganisms that are intended to have health benefits when consumed or applied to the body [15]. Although the mechanisms have not yet been fully elucidated, probiotics can cause modification of gut microbiota, enhancement of the epithelial barrier, increased adhesion to intestinal mucosa, inhibition of pathogen adhesion, and modulation of the immune system [16,17]. Probiotics are associated with the maintenance of healthy gut function, improved tolerance to antibiotics, glucose homeostasis and lipid metabolism, and overall reduced risk of various chronic diseases [18]. In a systemic review that included 70 studies, specific probiotics reportedly relieved lower gastrointestinal symptoms in irritable bowel syndrome, prevented diarrhea associated with antibiotics and *Helicobacter pylori* eradication therapy, and showed favorable safety [19]. In a recently updated guideline, probiotics were recommended for prevention of *Clostridium difficile* infection, acute infectious gastroenteritis in children, and prevention of necrotizing enterocolitis in preterm and low-birth-weight infants [20]. A recently published study showed that treatment of participants with a multispecies probiotic formulation significantly decreased the number of days of constipation and reduced abdominal pain/discomfort. In addition, there were differences in alpha diversity in the probiotic group compared to the placebo group [21]. In the present study, the beneficial effects of probiotic pretreatment on the gut microbiota and possible complications after bowel preparation for colonoscopy were investigated; results showed consuming probiotics before bowel preparation helped maintain the microbial diversity and recover the changed microbiota without missing taxa after bowel preparation. In addition, the duration of complications that occurred after bowel preparation in the active group was shorter than in the placebo group, and probiotic pretreatment was a significant factor in lowering the duration of minor complications. These results indicate that probiotics play an important role in the alteration and restoration of the gut microbiota after bowel preparation as well as in the minor complications that occur after colonoscopy.

In a randomized controlled trial in which the effects of preoperative bowel preparation were evaluated in patients undergoing colorectal surgery, a significant reduction was observed in the total number of bacteria [22]. In addition, the 16 rRNA gene sequence analysis of mucosal biopsies during sigmoidoscopy before and after bowel preparation showed that colonic lavage alters the microbial composition and diversity in the intestinal lumen and mucosa [23]. In a recent study, bowel preparation was suggested as a risk factor for postoperative delirium because it alters the gut microbial composition [24]. The results of the present study showed that the gut microbial diversity decreased after bowel preparation and similar microbiota reduction was observed in both groups, indicating that this was due to the bowel preparation. In addition, several taxa were equally increased after bowel preparation and then decreased in both groups; however, some taxa were increased only in the active group after bowel preparation, and subsequently, the gut microbiota were restored to almost the same level as before bowel preparation. *Veillonella*, an increased microbiota in the active group after bowel preparation, takes up lactate produced by *Lactobacillus* from the growth environment [25]. In addition, the anti-inflammatory species *Oribacterium* was increased only in the active group [26]. Although further studies are needed to confirm the results of this study, the data indicate that the microbiota that equally increased and decreased after bowel preparation could play a crucial role as key microbiota in early colonization after bowel preparation, and that probiotics may aid in the early colonization of key microbiota after bowel preparation.

The strength of the present study was the randomized, double-blind, placebo-controlled trial design in which the beneficial effects of probiotic pretreatment on the gut microbiota and the complications after bowel preparation for colonoscopy were investigated. The present study also had several limitations. First, the number of participants in this study was small. At the start of this study, we wanted to enroll 100 participants based on a previous study; however, this was not possible likely due to the age restriction which was not applied in the previous study. Interim analysis was performed after collecting feces from 32 participants and the data were nearly similar to the data from 51 participants. Similar results were expected even if the number of participants was increased; therefore, we considered that 51 participants were sufficient for analysis. Second, the mean age of participants in this study was relatively young (53.7 ± 1.1 years) compared to the average age of people undergoing bowel preparation for colonoscopy. We set the age of participants to 40–65 years to limit the age factor from acting as a variable, although it should be considered that complications are more prevalent in older people. Third, the association between the specific gut microbiota and the occurrence of complications after colonoscopy was not evaluated. Further studies are needed to confirm the results of this study. Fourth, this trial included only Korean participants and was conducted in a single institution. Therefore, the results may not be directly generalized to populations of other institutions and countries.

## 5. Conclusions

In conclusion, probiotic pretreatment had beneficial effects on the alteration and recovery of gut microbiota and possible complications after bowel preparation. Probiotics reduced the changes in microbial diversity, evenness, and distribution after bowel preparation and restored the gut microbiota several days after bowel preparation compared to the placebo. In addition, the pretreatment helped to decrease the duration of minor complications after bowel preparation. We also identified several strains including *Veillonella*, *Fusobacterium*, *Enterococcus*, *Eisenbergiella*, *Oribacterium*, *Parvimonas*, *Pepstreptococcus*, and *Holdemania* assumed as key microbiota in early colonization after bowel preparation, and it is posited that probiotics aid with this early colonization. Although the mechanism of probiotic effects on the microbial community is unclear, our study provides evidence of the role of probiotics in human health and disease.

## Figures and Tables

**Table 1:** Baseline characteristics and colonoscopy outcomes.

	Overall (*n* = 51)	Active (*n* = 26)	Placebo (*n* = 25)	*p*-Value
Age (years)	53.9 ± 8.0	54.4 ± 7.8	53.1 ± 8.3	0.589
Sex				0.773
Male	19 (37.3)	9 (34.6)	10 (40.0)	
Female	32 (62.7)	17 (65.4)	15 (60.0)	
Height (cm)	163.1 ± 9.0	162.7 ± 8.9	163.5 ± 9.3	0.771
Weight (kg)	66.6 ± 14.1	66.6 ± 13.1	66.7 ± 15.3	0.989
BMI (kg/m^2^)	24.9 ± 4.0	25.0 ± 3.7	24.8 ± 4.3	0.798
Alcohol history	15 (29.4)	7 (26.9)	8 (32.0)	0.894
Current smoker	6 (11.8)	4 (15.3)	2 (8.0)	0.674
Co-morbidities				0.450
Diabetes mellitus	3 (5.9)	1 (3.8)	2 (8.0)	
Hypertension	11 (21.6)	6 (23.2)	5 (20.0)	
Dyslipidemia	3 (5.9)	0 (0.0)	3 (12.0)	
Ischemic heart disease	2 (3.9)	1 (3.8)	1 (4.0)	
Dietary habits				0.892
Vegetarian	10 (19.6)	6 (23.2)	4 (16.0)	
Meat eater ^†^	4 (7.8)	2 (7.7)	2 (8.0)	
Eat evenly ^¶^	37 (72.6)	18 (69.1)	19 (76.0)	
Stool frequency				0.740
Low (≤2 times/week)	2 (3.9)	2 (7.7)	0 (0.0)	
Normal (1–2 times/1–2 days)	47 (92.2)	23 (88.5)	24 (96.0)	
High (≥3 times/day)	2 (3.9)	1 (3.8)	1 (4.0)	
Stool consistency (Bristol stool scale)				1.0
1–2	2 (3.9)	1 (3.8)	1 (4.0)	
3–5	47 (92.2)	24 (92.4)	23 (92.0)	
6–7	2 (3.9)	1 (3.8)	1 (4.0)	
Reason for colonoscopy				1.0
Screening	48 (94.1)	24 (92.3)	24 (96.0)	
Post-polypectomy surveillance	3 (5.9)	2 (7.7)	1 (4.0)	
Previous experience of colonoscopy	20 (39.2)	10 (38.5)	10 (40.0)	0.910
Previous experience of polypectomy	43 (84.3)	22 (84.6)	21 (84.0)	1.0
Bowel preparation				1.0
Good/fair	48 (94.1)	24 (92.3)	24 (96.0)	
Poor	3 (5.9)	2 (7.7)	1 (4.0)	
Findings of colonoscopy				0.902
Normal	12 (23.5)	6 (23.1)	6 (20.0)	
Polyp	32 (62.8)	17 (65.4)	15 (60.0)	
Cancer	0 (0.0)	0 (0.0)	0 (0.0)	
Diverticulosis	2 (3.9)	1 (3.8)	1 (4.0)	
Hemorrhoid	5 (9.8)	2 (7.7)	3 (12.0)	
Colon polyp				
Size (≥1 cm)	2 (3.9)	0 (0.0)	2 (11.8)	0.485
Resection				0.776
Cold forceps polypectomy	23 (45.1)	13 (50.0)	11 (44.0)	
Cold snare polypectomy	8 (15.7)	4 (15.4)	4 (16.0)	

Data are presented as the mean ± SD or number (percentage), BMI, body mass index. ^†^ Person with a meat-based diet. ^¶^ Person with a balanced diet of meat and vegetables.

**Table 2:** Post-colonoscopy complications.

	Overall (*n* = 51)	Active (*n* = 26)	Placebo (*n* = 25)	*p*-Value
Decreased body weight after bowel preparation (≥2 kg)	11 (21.6)	7 (26.9)	4 (16.0)	0.499
Post-colonoscopy complications	17 (33.3)	9 (34.6)	8 (32.0)	0.843
Type				0.783
Abdominal pain	4 (7.8)	2 (7.7)	2 (8.0)	
Abdominal pain/Bloating	2 (3.9)	1 (3.8)	1 (4.0)	
Bloating	8 (15.7)	5 (19.2)	3 (12.0)	
Bloating/Constipation	1 (2.0)	1 (3.8)	0 (0.0)	
Constipation	0 (3.9)	0 (0.0)	2 (8.0)	
Hemorrhage	0 (0.0)	0 (0.0)	0 (0.0)	
Perforation	0 (0.0)	0 (0.0)	0 (0.0)	
Onset timing				0.694
Day of colonoscopy	12 (23.5)	6 (23.1)	6 (24.0)	
1st day after colonoscopy	4 (7.8)	3 (11.5)	1 (4.0)	
2nd day after colonoscopy	0 (0.0)	0 (0.0)	0 (0.0)	
3rd day after colonoscopy	1 (2.0)	0 (0.0)	1 (4.0)	
Frequency				0.835
1 time/day	6 (11.8)	3 (11.5)	3 (12.0)	
2 times/day	6 (11.8)	4 (15.4)	2 (8.0)	
≥3 times/day	5 (9.8)	2 (7.7)	3 (12.0)	
Duration				0.025
<30 min	8 (15.7)	7 (26.9)	1 (4.0)	
1–2 h	4 (7.8)	1 (3.8)	3 (12.0)	
≥24 h	5 (9.8)	1 (3.8)	4 (16.0)	
Severity score (VAS)	3.6 ± 2.2	3.1 ± 1.6	4.1 ± 2.6	0.349

Data are presented as the mean ± SD or number (percentage). VAS, visual analogue scale (0: none, 10: very severe). Because the complications showed a significant difference based on the duration between the two groups, the duration (≥1–2 h) was set as a dependent variable in the analysis. In multivariate analysis, consuming probiotics before bowel preparation was identified as a significant factor in lowering the duration of minor complications (Table 3).

**Table 3:** Factors associated with complications (duration ≥ 1–2 h) after colonoscopy.

	Univariate Analysis			Multivariate Analysis		
Factor	OR	95% CI	*p*-Value	OR	95% CI	*p*-Value
Age						
<55 years	1					
≥55 years	0.57	0.13–2.43	0.444			
Sex						
Male	1					
Female	1.15	0.25–5.30	0.854			
BMI						
<25	1					
≥25	0.27	0.05–1.47	0.130			
Dietary habit						
Eat evenly	1					
Vegetarian	1.48	0.24–8.92	0.671			
Meat eater	1.72	0.15–19.49	0.661			
Co-morbidities						
None	1					
Present	0.30	0.56–1.62	0.162			
Polyp removal	0.72	0.17–3.11	0.661			
Group						
Placebo	1					
Active	0.22	0.04–1.21	0.082	0.13	0.02–0.69	0.027

OR, odds ratio; CI, confidence interval; BMI, body mass index.

## Data Availability

The data that support the findings of this study are available at https://www.ncbi.nlm.nih.gov/bioproject/PRJNA865884 (accessed on 4 August 2022), reference number PRJNA865884, and within the article and its Supplementary Materials.

## Supplementary Materials

The following supporting information can be downloaded at: https://www.mdpi.com/article/10.3390/nu15051141/s1, Figure S1. Comparative analysis via linear discriminant analysis (LDA) effect size (LEfSe) of each group at the family level (A, B, C). Figure S2. Comparative analysis of the 2nd and 4th visit groups via linear discriminant analysis (LDA) effect size (LEfSe) at the genus level.

## References

[1] Inadomi J.M., Vijan S., Janz N.K., Fagerlin A., Thomas J.P., Lin Y.V., Munoz R., Lau C., Somsouk M., El-Nachef N. (2012). Adherence to colorectal cancer screening: A randomized clinical trial of competing strategies. Arch. Intern. Med..

[2] van der Vlugt M., Grobbee E.J., Bossuyt P.M., Bongers E., Spijker W., Kuipers E.J., Lansdorp-Vogelaar I., Essink-Bot M.L., Spaander M.C., Dekker E. (2017). Adherence to colorectal cancer screening: Four rounds of faecal immunochemical test-based screening. Br. J. Cancer.

[3] Denberg T.D., Melhado T.V., Coombes J.M., Beaty B.L., Berman K., Byers T.E., Marcus A.C., Steiner J.F., Ahnen D.J. (2005). Predictors of nonadherence to screening colonoscopy. J. Gen. Intern. Med..

[4] Fisher D.A., Maple J.T., Ben-Menachem T., Cash B.D., Decker G.A., Early D.S., Evans J.A., Fanelli R.D., Fukami N., Hwang J.H. (2011). Complications of colonoscopy. Gastrointest. Endosc..

[5] Ko C.W., Riffle S., Shapiro J.A., Saunders M.D., Lee S.D., Tung B.Y., Kuver R., Larson A.M., Kowdley K.V., Kimmey M.B. (2007). Incidence of minor complications and time lost from normal activities after screening or surveillance colonoscopy. Gastrointest. Endosc..

[6] Cho H.S., Han D.S., Park H.S., Ahn S.B., Byun T.J., Kim T.Y., Eun C.S., Jeon Y.C., Sohn J.H. (2009). The incidence of minor complications and patients’ time requirements for colonoscopy. Korean J. Gastrointest. Endosc..

[7] Collatuzzo G., Boffetta P., Radaelli F., Cadoni S., Hassan C., Frazzoni L., Anderloni A., Laterza L., La Marca M., Rogai F. (2022). Incidence, risk and protective factors of symptoms after colonoscopy. Dig. Liver Dis..

[8] Drago L., Toscano M., De Grandi R., Casini V., Pace F. (2016). Persisting changes of intestinal microbiota after bowel lavage and colonoscopy. Eur. J. Gastroenterol. Hepatol..

[9] Harrell L., Wang Y., Antonopoulos D., Young V., Lichtenstein L., Huang Y., Hanauer S., Chang E. (2012). Standard colonic lavage alters the natural state of mucosal-associated microbiota in the human colon. PLoS ONE.

[10] Nagata N., Tohya M., Fukuda S., Suda W., Nishijima S., Takeuchi F., Ohsugi M., Tsujimoto T., Nakamura T., Shimomura A. (2019). Effects of bowel preparation on the human gut microbiome and metabolome. Sci. Rep..

[11] O’Brien C.L., Allison G.E., Grimpen F., Pavli P. (2013). Impact of colonoscopy bowel preparation on intestinal microbiota. PLoS ONE.

[12] Jalanka J., Salonen A., Salojarvi J., Ritari J., Immonen O., Marciani L., Gowland P., Hoad C., Garsed K., Lam C. (2015). Effects of bowel cleansing on the intestinal microbiota. Gut.

[13] Kim J.H., Choi Y.J., Kwon H.J., Jung K., Kim S.E., Moon W., Park M.I., Park S.J. (2021). Effect of gut microbiome on minor complications after a colonoscopy. Intest. Res..

[14] Kim J.H. The Change of Gut Microbiota after Bowel Preparation and the Effect of Probiotics: A Randomized Controlled, Open-Label Trial. Identifier: NCT03760133. Updated 20 February 2020. NCT03760133.

[15] Hotel A.C.P., Cordoba A. (2001). Health and nutritional properties of probiotics in food including powder milk with live lactic acid bacteria. Prevention.

[16] Bermudez-Brito M., Plaza-Díaz J., Muñoz-Quezada S., Gómez-Llorente C., Gil A. (2012). Probiotic mechanisms of action. Ann. Nutr. Metab..

[17] Wieërs G., Belkhir L., Enaud R., Leclercq S., Philippart de Foy J.M., Dequenne I., de Timary P., Cani P.D. (2019). How Probiotics Affect the Microbiota. Front. Cell Infect. Microbiol..

[18] Sanders M.E., Lenoir-Wijnkoop I., Salminen S., Merenstein D.J., Gibson G.R., Petschow B.W., Nieuwdorp M., Tancredi D.J., Cifelli C.J., Jacques P. (2014). Probiotics and prebiotics: Prospects for public health and nutritional recommendations. Ann. N. Y. Acad. Sci..

[19] Hungin A.P.S., Mitchell C.R., Whorwell P., Mulligan C., Cole O., Agréus L., Fracasso P., Lionis C., Mendive J., Philippart de Foy J.M. (2018). Systematic review: Probiotics in the management of lower gastrointestinal symptoms—An updated evidence-based international consensus. Aliment. Pharmacol. Ther..

[20] Su G.L., Ko C.W., Bercik P., Falck-Ytter Y., Sultan S., Weizman A.V., Morgan R.L. (2020). AGA Clinical Practice Guidelines on the Role of Probiotics in the Management of Gastrointestinal Disorders. Gastroenterology.

[21] Labenz J., Borkenstein D.P., Heil F.J., Madisch A., Tappe U., Schmidt H., Terjung B., Klymiuk I., Horvath A., Gross M. (2023). Application of a multispecies probiotic reduces gastro-intestinal discomfort and induces microbial changes after colonoscopy. Front. Oncol..

[22] Watanabe M., Murakami M., Nakao K., Asahara T., Nomoto K., Tsunoda A. (2010). Randomized clinical trial of the influence of mechanical bowel preparation on faecal microflora in patients undergoing colonic cancer resection. Br. J. Surg..

[23] Ringel Y., Maharshak N., Ringel-Kulka T., Wolber E.A., Sartor R.B., Carroll I.M. (2015). High throughput sequencing reveals distinct microbial populations within the mucosal and luminal niches in healthy individuals. Gut Microbes.

[24] Yang Z., Tong C., Qian X., Wang H., Wang Y. (2022). Mechanical Bowel Preparation Is a Risk Factor for Postoperative Delirium as It Alters the Gut Microbiota Composition: A Prospective Randomized Single-Center Study. Front. Aging Neurosci..

[25] Scheiman J., Luber J.M., Chavkin T.A., MacDonald T., Tung A., Pham L.-D., Wibowo M.C., Wurth R.C., Punthambaker S., Tierney B.T. (2019). Meta-omics analysis of elite athletes identifies a performance-enhancing microbe that functions via lactate metabolism. Nat. Med..

[26] Torres-Maravilla E., Boucard A.-S., Mohseni A.H., Taghinezhad-S S., Cortes-Perez N.G., Bermúdez-Humarán L.G. (2021). Role of gut microbiota and probiotics in colorectal cancer: Onset and progression. Microorganisms.

