# Supplementary material for: Benefits of Probiotic Pretreatment on the Gut Microbiota and Minor Complications after Bowel Preparation for Colonoscopy: A Randomized Double-Blind, Placebo-Controlled Pilot Trial
Source: Nutrients. 2023 Feb 24;15(5):1141. doi: 10.3390/nu15051141 (PMC10004836; doi:10.3390/nu15051141)
Supplement: Supplementary file 1 [file nutrients-15-01141-s001.zip › Questionnaire 1 (Supplementary data).pdf]

Questionnaire 1 (1 month before colonoscopy)

Subject code (   )

1. Gender ( ) Male ( ) Female
2. Age (   )
3. Height / Weight (/   )
4. Alcohol ( ) none ( ) past ( ) current
5. Smoking ( ) none ( ) past ( ) current
6. Comorbid diseases

   ( ) Diabetes ( ) Hypertension ( ) Hyperlipidemia

   ( ) Ischemic heart disorder

   * Others (describe it yourself) (   )
7. Reason for colonoscopy

   ( ) Screening ( ) Post-polypectomy surveillance

   ( ) Have any symptoms ( ) Positive fecal occult blood test
8. Do you have any symptoms last one month?

   ( ) No ( ) Yes
9. If you had any symptoms, what was it?

   ( ) Abdominal pain ( ) Bloating ( ) Constipation ( ) Diarrhea

   ( ) Nausea ( ) Vomiting ( ) Anal bleeding ( )

   * Others (describe it yourself) (   )
10. Previous colonoscopy experience

    ( ) No ( ) One to Two times ( ) More than three times
11. Dietary habits

    ( ) Vegetarian ( ) Meat eater ( ) Eat evenly
12. Number of defecations per day or week

    (/ per day) or (/ per week)
13. What is your usual form of feces?

    (Bristol stool scale)

    ( ) type 1 ( ) type 2 ( ) type 3 ( ) type 4

    ( ) type 5 ( ) type 6 ( ) type 7
