# Supplementary material for: Benefits of Probiotic Pretreatment on the Gut Microbiota and Minor Complications after Bowel Preparation for Colonoscopy: A Randomized Double-Blind, Placebo-Controlled Pilot Trial
Source: Nutrients. 2023 Feb 24;15(5):1141. doi: 10.3390/nu15051141 (PMC10004836; doi:10.3390/nu15051141)

Questionnaire 2 (7th day after colonoscopy)

Subject code (   )

1. What was the form of your feces for the first time after colonoscopy? (Bristol stool scale)
   ( ) type 1 ( ) type 2 ( ) type 3 ( ) type 4 ( ) type 5 ( ) type 6 ( ) type 7
2. What was the form of your feces after 7 days of colonoscopy? (Bristol stool scale)
   ( ) type 1 ( ) type 2 ( ) type 3 ( ) type 4 ( ) type 5 ( ) type 6 ( ) type 7
3. Did you have any symptoms after colonoscopy?
   ( ) No ( ) Yes
4. If you had any symptoms, what was it?
   ( ) Abdominal pain ( ) Bloating ( ) Constipation ( ) Diarrhea
   ( ) Nausea ( ) Vomiting ( ) Cold sweating ( ) Bleeding
5. When did it start after colonoscopy?
   ( ) On the day ( ) One day after ( ) Two days after
   ( ) Three days after ( ) Four days after ( ) Five days after
   ( ) Six days after ( ) Seven days after
6. How often did it happen?
   ( ) One a day ( ) Twice a day
   ( ) More than three times a day(   )
7. How long did it last?
   ( ) Less than 30 minutes ( ) 1 to 2 hours ( ) 2 to 6 hours
   ( ) 6 to 12 hours ( ) 12 to 24 hours ( ) More than 24 hours
8. What is the score of the symptom?
   (   )

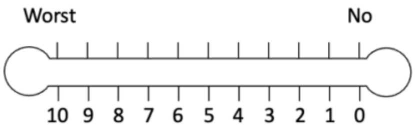

Supplement: Supplementary file 1 [file nutrients-15-01141-s001.zip › Questionnaire 2 (Supplementary data).pdf]
