# Supplementary material for: Benefits of Probiotic Pretreatment on the Gut Microbiota and Minor Complications after Bowel Preparation for Colonoscopy: A Randomized Double-Blind, Placebo-Controlled Pilot Trial
Source: Nutrients. 2023 Feb 24;15(5):1141. doi: 10.3390/nu15051141 (PMC10004836; doi:10.3390/nu15051141)
Supplement: Supplementary file 1 [file nutrients-15-01141-s001.zip › SupplementaryFigures.pdf]

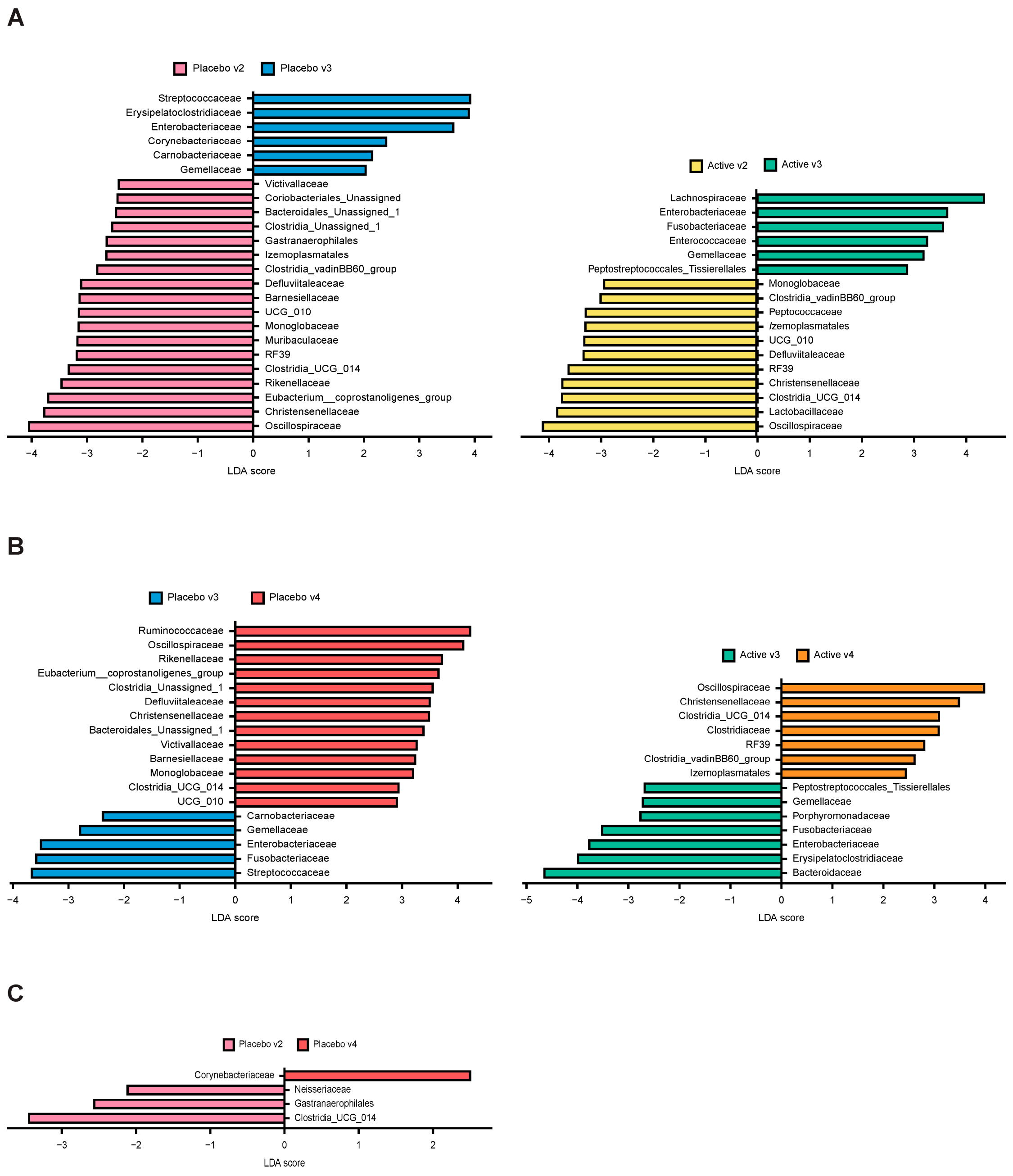

Figure S1. Comparative analysis via linear discriminant analysis (LDA) effect size (LEfSe) of each group at the family level (A, B, C).

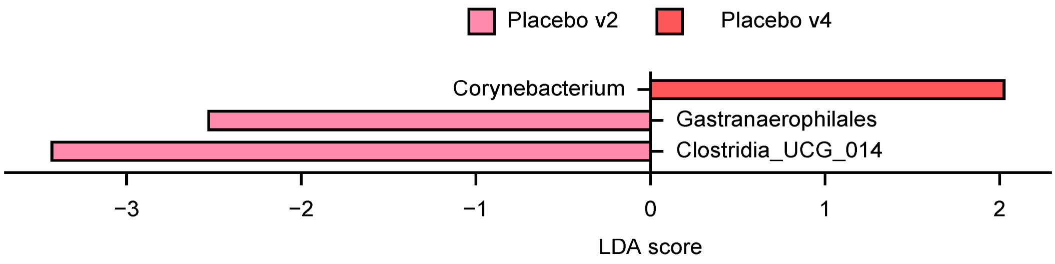

Figure S2. Comparative analysis of the 2nd and 4th visit groups via linear discriminant analysis (LDA) effect size (LEfSe) at the genus level.
